# Effects of laboratory salmon louse infection on osmoregulation, growth and survival in Atlantic salmon

**DOI:** 10.1093/conphys/coaa023

**Published:** 2020-03-26

**Authors:** Per Gunnar Fjelldal, Tom J Hansen, Ørjan Karlsen

**Affiliations:** 1 Reproduction and Developmental Biology, Institute of Marine Research (IMR), Matre Aquaculture Research Station, 5984 Matredal, Norway; 2 Reproduction and Developmental Biology, Institute of Marine Research (IMR), PO Box 1870, Nordnes, 5817 Bergen, Norway

**Keywords:** Atlantic salmon, chloride, cortisol, infection intensity, salmon louse, sodium

## Abstract

Anadromous Atlantic salmon (*Salmo salar*) rely on long ocean migrations to build energy stores for maturation and spawning. In seawater, wild Atlantic salmon are threatened by high salmon lice (*Lepeophtheirus salmonis*) infestation levels resulting from intensive salmonid sea-cage aquaculture. Salmon lice infection can cause a stress response and an osmotic imbalance in the host. The lice infection intensity threshold values for these responses, however, remain to be identified in Atlantic salmon. In order to define this under laboratory conditions, individually tagged F1 wild origin Atlantic post-smolts (40 g) were infected with salmon lice copepodids or left as uninfected controls. Twenty-eight days post infection, infected post-smolts had a mean of 0.38 (range of 0.07–0.9) mobile lice g^−1^ fish weight. During this period, specific growth rates (SGRs) were lower in infected than control fish (0.4 vs 1.0% day^−1^). Higher plasma Na^+^, Cl^−^ and osmolality in infected fish also indicate osmoregulatory impairment. SGR correlated negatively with plasma Na^+^, Cl^−^, osmolality and cortisol in the infected, but not in the control group. Infection intensity (lice g^−1^ fish) correlated positively with mortality rate and plasma Na^+^, Cl^−^, osmolality and cortisol and correlated negatively with SGR and condition factor. Calculated lice intensity threshold values for changes in plasma ions were 0.18 lice g^−1^ for plasma Cl^−^, and 0.22 lice g^−1^ for plasma Na^+^. Moribund infected fish occurred at infection intensities above 0.2 lice g^−1^, and these fish had extreme plasma Cl^−^, Na^+^, osmolality and cortisol levels. There was a positive correlation between plasma cortisol and plasma Na^+^, Cl^−^ and osmolality in infected fish. This study provides vital information that can be used to define thresholds in the monitoring and conservation of wild Atlantic salmon populations affected by aquaculture-driven salmon lice infestations.

## Introduction

Anadromous Atlantic salmon smolts migrate to the sea during the spring to grow and enter puberty and finally migrate back to their river of origin to complete sexual maturation and spawn (Klemetsen *et al*., 2003). Normal smolt size for wild Atlantic salmon smolts is 10 to 80 g (Thorstad *et al*., 2011). During their natural outward migration, wild Atlantic salmon post-smolts can get infected by the parasitic copepod salmon louse (*Lepeophtheirus salmonis*), with salmon sea-cage aquaculture increasing the severity of impact by elevating the infection level (Costello, 2009; Taranger *et al*., 2015; Forseth *et al*., 2017). Unlike sea trout (*Salmo trutta*) (reviewed in Thorstad *et al*., 2015), Atlantic salmon do not return prematurely to freshwater to shed salmon lice, but stay in seawater for a minimum of 1 year. Anadromous Arctic char (*Salvelinus alpinus*), on the other hand stay in seawater for only 1–2 months (Jørgensen and Johnsen, 2014). Hence, compared to the other endemic anadromous salmonids in the North Atlantic, Atlantic salmon may be especially vulnerable if salmon lice infection level is high.

In seawater, fish actively excrete Na^+^ and Cl^−^ to maintain ionic balance (Marshall, 2002). Salmon lice damage the host skin, mucus surface and dermal tissue, damaging the barrier between seawater and the fish body, threatening ionic balance (Bjørn and Finstad, 1997). In addition, salmon lice infection elevates plasma cortisol (Grimnes and Jakobsen, 1996; Wells *et al*., 2006; Wells *et al*., 2007; Tveiten *et al*., 2010), a stress hormone that increases epithelial membrane permeability (Bonga, 1997). Thereby, Atlantic salmon infected by salmon lice can show elevated plasma Na^+^, Cl^−^ and osmolality (Grimnes and Jakobsen, 1996; Bowers *et al*., 2000; Finstad *et al*., 2000), and this is a root cause for lice induced mortality (Grimnes and Jakobsen, 1996; Finstad *et al*., 2000). Grimnes and Jakobsen (1996) studied the physiological response to laboratory lice infection in 40 g Atlantic salmon post smolts and observed that a mean infection intensity of ~ 1.6 (lice g^−1^ fish) caused elevated plasma Cl^−^ and an exponential increase in mortality 25 days post infection. Based on the results, the authors suggested that an infection intensity of above ~ 0.6 lice g^−1^ appear to cause death of Atlantic salmon post-smolts after the lice have reached the pre-adult stage. Further, Grimnes and Jakobsen (1996) found a positive correlation between infection density (lice g cm^−3^) and plasma Cl^−^, but the data were based on infection intensities all above ~ 0.6. Finstad *et al*. (2000) did not find a correlation between plasma Cl^−^ and infection intensity in a laboratory lice infection experiment with 60 g first generation wild Atlantic salmon post-smolts and a mean infection intensity of ~ 0.6, but reported higher levels in infected compared to uninfected fish as the lice matured on the fish. Bowers *et al*. (2000) used 680 g Atlantic and a mean infection intensity of ~ 0.16 and found no effect of lice infection on plasma electrolyte levels, when compared to uninfected control. Laboratory salmon lice infection intensities above 0.3 impact on osmoregulatory physiology in post-smolt Arctic char (Fjelldal *et al*., 2019). Wells *et al*. (2006) studied the sublethal threshold burden of salmon lice in sea trout postsmolts under laboratory conditions, and found that 13 mobile lice per fish in fish ranging between 19 and 70 g was a breakpoint across several physiological parameters (glucose, lactate, chloride). Such knowledge is lacking in Atlantic salmon, where the physiological effects of the sea lice *Caligus rogercresseyi* have been extensively studied (Gonzalez *et al*., 2015; 2016a; 2016b; 2016c). However, *Lepeophtheirus salmonis* is the sea lice species that concerns the conservation of wild Atlantic salmon. In Norway, rates of mortality or premature return to freshwater in wild salmonids is estimated based on the registered *Lepeophtheirus salmonis* infection intensity (Taranger *et al*. 2015). This classification system—the salmon lice risk index—parameterizes a national-scale model used to quantify the risk of lice-induced mortality in wild Atlantic salmon and regulate the Norwegian salmon farming industry (Kristoffersen *et al*. 2018). Providing threshold values for physiological consequences of *Lepeophtheirus salmonis* in Atlantic salmon would broaden the scientific base for the conservation of anadromous salmonids in the North Atlantic.

In order to explore the lice infection intensity threshold for osmotic imbalance and stress response in Atlantic salmon post-smolts, first generation ~ 40 g wild Atlantic salmon post-smolts were infected with salmon lice copepodids at a mean infection intensity of 0.38 (min 0.07, max 0.9) lice g^−1^. Uninfected fish served as control. The study was terminated 28 days post infection. Response parameters monitored during the experiment were mortality, growth rate, condition factor, and plasma osmolality, Na^+^, Cl^−^ and cortisol.

## Material and methods

The Atlantic salmon (*Salmo salar*) used in the present experiment were first generation wild fish brought to Institute of Marine Research, as eyed eggs in January 2017, originating from wild caught Atlantic salmon (male and female) from River Etne, Hordaland county, Western Norway. First feeding was in April 2017. Then, the fish were reared under continuous light and a constant temperature of 12°C until summer solstice, when water temperature was changed to ambient. Photoperiod was changed from continuous light to simulated natural photoperiod (Western Norway, 60° N, 5° E) in October 2017. On 21 December 2017, 330 fish were PIT-tagged (Glass tag 2, 12 mm, TrackID AS, Stavanger, Norway) and randomly distributed in four square white covered fiberglass tanks (1 × 1 × 0.43 m), with 82 fish in each. In the period 21 December 2017 to 25 January 2018, the fish were reared under continuous light and 10°C. On 25 January 2018, water salinity was increased to 28 ppt and temperature decreased to 9°C. The fish were at these conditions until 12 February 2018, when salinity was increased to 34 ppt with temperature unchanged. On 19 February, the fish were redistributed to the experimental tanks (same tank type as before).

### Ethical statement

All experiments were performed at the Institute of Marine Research, Matre Research Station (60° N, 5° E, Western Norway), which is authorized for animal experimentation (Norwegian Food Safety Authority, facility 110), in accordance with International guidelines certified using Norwegian research permit number 14982.

### Experimental setup

On 19 February 2018 (day 0), 330 salmon post-smolts were anaesthetized (Finquel, 0.1 g L^−1^), measured for fork length and body weight and randomly distributed between six square white covered fiberglass tanks (1 × 1 × 0.43 m) (*n* = 55 per tank). On 21 February (day 2), three of the tanks were infected with salmon lice (*L. salmonis*) copepodids, while three tanks were uninfected controls. In all six tanks (3 infected, 3 uninfected), the water level was reduced to 10 cm depth, and water flow was stopped before adding copepodids (10 days post-hatch) to the three infection tanks. Then, in all tanks, the water flow (normal) was turned back after 20 min. In total, 4290 copepodids were used to infect the fish (1430 copepodids per tank), giving an average infection pressure of 26 lice per fish. The experiment was terminated 28 days post-infection (day 30, 21 March 2018). The fish were anaesthetized (0.01 g L^−1^, Aquacalm vet., Scan Aqua AS, Årnes, Norway) followed by reading the PIT tag, measuring fork length and body weight and counting lice. After counting lice, the fish were killed by a blow to the head. Counts of lice per fish included all lice remaining in individual anaesthetic water containers they were place in, in addition to those on live and dead fish. By the time of sampling, mobile pre-adult II male and pre-adult I and II female stages had developed at 9°C (Hamre *et al*., 2019). Only lice number rather and not the stage was quantified. After recording the PIT tag, measuring length and weight (all six tanks, lice infected and control), and counting lice (3 lice infected tanks), blood was collected from 15 fish per tank (45 per group, all six tanks, lice infected and control). In the infected tanks, in total 10 fish were moribund. These 10 moribund fish and 35 random fish (normal behaviour) were sampled for blood in the infected group. There were no moribund fish in the control tanks, and 45 random fish were sampled for blood in this group. Blood was centrifuged and plasma stored at −80°C until analysis. Blood sample was taken from the caudal vessel with a heparinized tuberculin syringe fitted with a 25-gauge needle.

Lice for the infection were produced from an outbred strain that had been maintained at ~9°C at the Institute of Marine Research lice hatchery using methods described in Hamre *et al*. (2009).

In total, 4290 copepodids were used to infect the present fish, and 2319 of these were attached at the day of counting lice. Twenty-nine lice infected fish that died during the experiment were not counted for lice, but based on 10 lice counted dead fish, the unregistered fish were estimated to have in total 638 attached salmon lice. Hence, in total 2957 attached lice were expected if all fish had survived until the terminal sampling, that is, 69% of the copepodids that were added during the infection. Likely, not all copepodids attached, and some may have died or dislodged after attachment (Wagner *et al*., 2008).

### Plasma analysis

Plasma ion levels (Na^+^, Cl^−^) were detected with an ABL90 FLEX PLUS blood gas analyzer (Radiometer Medical ApS, Åkandevej 21, DK-2700, Brønshøj, Denmark). Plasma osmolality was determined by freeze point determination (Fiske Micro Osmometer Model 210, Norwood, MA, USA).

Plasma cortisol concentration was quantified with an ELISA assay kit (IBL International GmbH) and a Sunrise microplate reader (Tecan).

### Calculations and statistical analysis

Infection intensity (II) was calculated using: *II* = *Ln Fw*^−1^, where *Ln* was number of lice on infected fish and *Fw* was body weight (g) of infected fish at time of counting lice on Day 30.

The condition factor (CF) was calculated using: *CF* = (*WL*^−3^)100, where *W* was the live body weight (g) and *L* was the fork length (cm). Specific growth rate was calculated using: SGR = (*e^G^* − 1)100, where *G* = (ln(*X*_2_) − ln (*X*_1_))/(*t*_2_ − *t*_1_), *X*_2_ and *X*_1_ were the body weights at times *t*_2_ and *t*_1_. Change in CF (ΔCF) was calculated using: ΔCF = CF_2_ − CF_1_, where CF_1_ was CF on sampling number 1, and *CF*_2_ was CF on sampling number 2.

**Figure 1:**
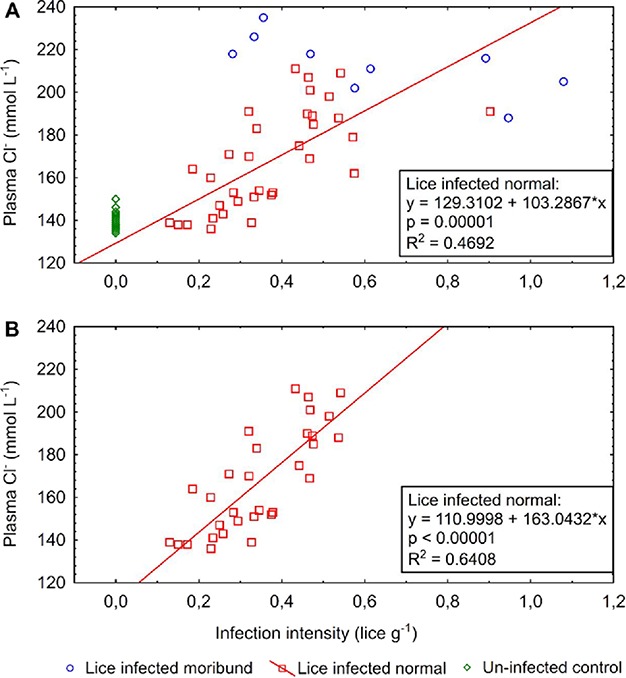
Simple regressions between infection intensity (lice g^−1^) and plasma Cl^−^ (mmol L^−1^). (**A**) Trend line, P and *R*^2^ values are based on data from the lice infected normal group. Data from lice infected moribund fish and the uninfected control group are included for comparison. (**B**) Data from lice infected normal fish with infection intensity < 0.55 lice g^−1^ (mean infection intensity in dead fish).

Lice infection intensity threshold values were calculated for plasma Cl^−^ and Na^+^. Plasma levels in the control group were used in order to calculate lice infection intensity thresholds for response in these plasma ions. For this purpose, we used the simple regression equations from the simple regression between infection intensity and plasma Na^+^ and Cl^−^ in the infected fish (*n* = 35; excluding moribund fish), and mean plus two standard deviations plasma Na^+^ and Cl^−^ values recorded in the control group (Table 8). For example, for calculation of infection intensity threshold (IIT) for response in plasma Cl^−^, *R*^2^ for the simple linear regression between lice infection intensity (II, x-axis) and plasma Cl^−^ (Cl^−^, y-axis) in infected fish was 0.47, and the simple linear regression equation was *y* = 129.3102 + 103.2867^*^x (Fig. 1A). Entering the mean plus two standard deviation plasma chloride values from the control group (147.5 mmol L^−1^) into the equation gives a calculated infection intensity threshold of 0.18 lice fish^−1^ for plasma Cl^−^.

The data were analysed using Statistica version 12 (StatSoft, Inc., 2300 East 14th Street, Tulsa, OK, USA). Results are shown as means with their standard errors. Data were tested for homogeneity in variance (Levene’s *F* test) and normality (Kolmogorov Smirnov test). Excluding moribund fish, significant differences within different plasma parameters (Na^+^, Cl^−^, osmolality and cortisol) between infected and control fish were tested by two-way nested ANOVAs with tank as random factor nested in treatment. Including moribund fish, significant differences within different plasma parameters between moribund infected, ‘normal’ infected and control fish were tested by Kruskal–Wallis ANOVAs. Significant differences in length, weight, CF, mm day^−1^, SGR and ΔCF were tested by two-way nested ANOVAs with tank as random factor nested in treatment (this analysis include moribund fish). Possible significant correlations between measured parameters were tested by product–moment and partial correlations. *P* value < 0.05 was considered statistically significant.

## Results

### Infection intensity Day 30

Mean Infection intensity (lice g^−1^) in the three infected tanks were 0.46 (± 0.029), 0.33 (± 0.027) and 0.36 (± 0.031), and 0.38 (± 0.017) overall. This equated to mean numbers of lice fish^−1^ of 20 (± 1.2), 15 (± 0.9) and 16 (± 1.1) in the three tanks and 17 (± 0.6) across all individuals, with 100% prevalence. There was no correlation between size on Day 0 and infection intensity on Day 30, 28 days post infection.

### Mortality

In total, 39 (23.6%) and 21 (12.7%) individuals died during the experimental period in the lice infected and control groups, respectively. In the control group, mortality caused by skin lesions occurred between Days no. 15 and 22 and were restricted to two tanks. Twelve (Tank 5) and 9 (Tank 6) fish died in these, with zero mortality in the last tank (Tank 4), where no skin lesions were observed. Inspection by veterinary followed by bacterial culture (kidney) reported that some type of *Vibrio* bacteria was expected to stand behind the mortalities, but a further classification of the bacteria type was not performed. After the mortality stopped in Tanks 5 and 6, no skin lesions were observed in these tanks. In the infected group, the mortalities were between Day nos. 22 and 30 and occurred in all three infected tanks at similar levels. Sixteen (Tank 7), 15 (Tank 8) and 8 (Tank 9) fish died in each tank between Days 22 and 30. Mortalities associated with skin lesions like those observed in control Tanks 5 and 6 were not observed in the infected tanks, nor were similar lesions observed in infected fish that survived. Between Day nos. 22 and 30, in total 10 random dead fish were counted for lice. For eight of these, length and weight were not recorded. The last two dead fish were registered on Day 30, at the terminal sampling, and were recorded for length and weight. Mean number of lice fish^−1^ on the dead fish was 22 ± 2.8 (*n* = 10), and infection intensity 0.55 ± 0.00 (*n* = 2). In addition to the registered dead fish, 10 infected fish (Tank 7, *n* = 5; Tank 8, *n* = 1; Tank 9, *n* = 4) were moribund at the terminal sampling on Day 30. Compared to infected fish with normal behaviour, moribund fish did not avoid the net when netted out for sedation and lice counting. Adding these to the dead fish, total mortality in the infected group was 29.7%. Moribund fish (*N* = 10, infection intensity 0.60 ± 0.09 lice g^−1^, 22 ± 3.5 lice fish^−1^) had significantly higher (Mann–Whitney *U* test, *P* = 0.0028) infection intensity compared to ‘normal’ infected fish (*N* = 116, infection intensity 0.37 ± 0.02 lice g^−1^, 16 ± 0.6 lice fish^−1^).

Fish that died during the experiment or survived had equal size on Day 0 (data not shown).

### Physiology 28 days post infection (Day 30)

Infected fish with normal behaviour had significantly higher (two-way nested ANOVA, *P* < 0.05) plasma Na^+^, Cl^−^ and osmolality than control fish (Table 1). Moribund infected fish had significantly higher (Kruskal–Wallis ANOVA, multiple comparison of mean ranks, *P* < 0.05) plasma Na^+^, Cl^−^, osmolality and cortisol than both ‘normal’ lice-infected and control fish (Table 1). There were significant relationships between plasma cortisol and plasma Na^+^, Cl^−^ and osmolality in lice infected fish and between plasma cortisol and plasma osmolality in uninfected control fish (Table 2).

**Table 1:** Plasma Na^+^, Cl^−^, osmolality and cortisol in Atlantic salmon (*Salmo salar*) on Day 30, 28 days post infection with salmon lice (*Lepeophtheirus salmonis*)

**Parameter**	**Salmon lice infected**		**Control**	***P* value** ^*^	***P* value** ^**^
	Normal	Moribund			
*N* per category	35	10	45		
Na^+^ (mmol L^−1^)	190.6 ± 3.8b	233.9 ± 4.9a	169.3 ± 0.5c	***0.0276***	***0.0000***
Cl^−^ (mmol L^−1^)	167.6 ± 3.9b	213.2 ± 4.6a	140.3 ± 0.5c	***0.0125***	***0.0000***
Osmolality (mOsm kg^−1^)	380.8 ± 7.2b	469.9 ± 10.9a	347.4 ± 1.8c	***0.0467***	***0.0000***
Cortisol (ng ml^−1^)	193.8 ± 27.4b	470.7 ± 26.7a	86.4 ± 10.0c	0.1826	***0.0000***

*N* = 45 per treatment group (15 per tank). Some of the infected fish were moribund at the terminal sampling.

^*^Number in italic and bold indicates a significant difference (two-way nested ANOVA, *P* < 0.05) between ‘Salmon lice infected Normal’ and ‘Control’ fish.

^**^Number in italic and bold indicates a significant difference (Kruskal–Wallis ANOVA, *P* < 0.05) between ‘Salmon lice infected Normal’, ‘Salmon lice infected Moribund’ and ‘Control’ fish. Different lowercase letters indicate significant differences.

**Table 2:** Coefficient of determination (*R*^2^) and *P* values for the relationships between plasma Na^+^, Cl^−^, osmolality and cortisol

**Correlation**	***R*** ^**2**^	***P* value**	***N***
*Control group*			
Na^+^ vs Cl^−^	0.47 (+)	***<0.00001***	45
Na^+^ vs Osm	0.16 (+)	***0.0068***	45
Cl^−^ vs Osm	0.05	0.1381	45
Cortisol vs Na^+^	<0.01	0.7339	45
Cortisol vs Cl^−^	<0.01	0.7267	45
Cortisol vs Osm	0.32 (+)	***0.0001***	45
*Infected group*			
Na^+^ vs Cl^−^	0.96 (+)	***<0.00001***	35^*^
Na^+^ vs Osm	0.63 (+)	***<0.00001***	35^*^
Cl^−^ vs Osm	0.63 (+)	***<0.00001***	35^*^
Cortisol vs Na^+^	0.74 (+)	***<0.00001***	35^*^
Cortisol vs Cl^−^	0.72 (+)	***<0.00001***	35^*^
Cortisol vs Osm	0.37 (+)	***0.0001***	35^*^

Na^+^ vs Cl^−^	0.98 (+)	***<0.00001***	45^**^
Na^+^ vs Osm	0.78 (+)	***<0.00001***	45^**^
Cl^−^ vs Osm	0.78 (+)	***<0.00001***	45^**^
Cortisol vs Na^+^	0.78 (+)	***<0.00001***	45^**^
Cortisol vs Cl^−^	0.78 (+)	***<0.00001***	45^**^
Cortisol vs Osm	0.54 (+)	***<0.00001***	45^**^

Number in italic and bold indicates a significant correlation. ‘+’ indicates a positive relationship, while ‘−’ indicates a negative relationship.

^*^Do not include values from moribund fish.

^**^Include values from moribund fish.

### Growth

No differences (two-way nested ANOVA, *P* > 0.6) in length, weight and condition factor (CF) were present between the treatment groups at the start of the experiment on day 0 (Table 3). On Day 30, lice infected fish had significantly lower length, weight and condition factor (Table 3). The calculated mm day^−1^, SGR and ΔCF in the period between Days 0 and 30 were significantly lower in the lice infected compared to the control group (Table 3).

**Table 3:** Fork length, body weight, condition factor on Days 0 and 30 and change in length (mm day^−1^), weight (SGR, % day^−1^) and condition factor (ΔCF) between Days 0 and 30 in Atlantic salmon (*Salmo salar*) that were infection with salmon lice (*Lepeophtheirus salmonis*) copepodites on Day 2

**Parameter**	**Salmon lice infected**	**Control**	***P* value** ^*^
*Day 0*			
Length (cm)	15.1 ± 0.1	15.1 ± 0.1	0.4674
Weight (g)	39.9 ± 0.5	39.2 ± 0.5	0.1222
Condition factor	1.15 ± 0.01	1.14 ± 0.01	0.1090
*Day 30*			
Length (cm)	15.8 ± 0.1	16.3 ± 0.1	***0.0011***
Weight (g)	44.9 ± 0.8	53.5 ± 0.8	***0.0013***
Condition factor	1.13 ± 0.01	1.22 ± 0.01	***0.0119***
mm day^−1^	0.23 ± 0.01	0.39 ± 0.01	***0.0007***
SGR	0.42 ± 0.04	0.95 ± 0.03	***<0.0001***
Delta ΔCF	−0.01 ± 0.01	0.07 ± 0.01	***0.0154***

Control *N* = 146 (43–55 per tank), Salmon lice infected *N* = 140 (44–50 per tank)

^*^Number in italic and bold indicates a significant difference (two-way nested ANOVA, *P* < 0.05).

There was a significant relationship between SGR and plasma Na^+^, Cl^−^, osmolality and cortisol in the infected group, but not in the uninfected control (Table 4).

**Table 4:** Coefficient of determination (*R*^2^) and *P* values for the relationships between SGR and plasma Na^+^, Cl^−^, osmolality and cortisol

**Correlation**	***R*** ^**2**^	***P* value**	***N***
*Control group*			
SGR vs Na	0.03	0.2383	45
SGR vs Cl	0.01	0.5559	45
SGR vs osmolality	<0.01	0.9404	45
SGR vs cortisol	<0.01	0.5753	45
*Infected group*			
SGR vs Na	0.61 (−)	***<0.00001***	35^*^
SGR vs Cl	0.65 (−)	***<0.00001***	35^*^
SGR vs osmolality	0.58 (−)	***<0.00001***	35^*^
SGR vs cortisol	0.48 (−)	***<0.00001***	35^*^
SGR vs Na	0.66 (−)	***<0.00001***	45^**^
SGR vs Cl	0.67 (−)	***<0.00001***	45^**^
SGR vs osmolality	0.60 (−)	***<0.00001***	45^**^
SGR vs cortisol	0.54 (−)	***<0.00001***	45^**^

Number in italic and bold indicates a significant correlation. ‘+’ indicates a positive relationship, while ‘−’ indicates a negative relationship

^*^Do not include values from moribund fish.

^**^Include values from moribund fish.

### Infection intensity thresholds

To visualize the burden of salmon lice infection on Atlantic salmon at different infection intensities, data on growth, mortality and physiology were categorized according to infection intensity, and mean values within infection intensity categories are shown in Tables 5 and 6. Mean lice fish^−1^ was 22 in both the moribund fish (Days 30, 10 fish) and dead fish (Days 22–30, 10 random dead fish) suggesting that combining the data on moribund and dead on Day 30 are representative for how infection intensity affected mortality at this stage (28 days post infection). For infected individuals, mortality (dead (*n* = 2) and moribund fish (*n* = 10)) at Day 30 was higher with elevated infection intensity (Table 5), with no mortalities occurring at < 0.2 lice g^−1^. SGR, ΔCF and CF were lower with higher infection intensity (Table 5), and fish with infection intensities ≥0.6 had negative SGR. Plasma Na^+^, Cl^−^, osmolality and cortisol were higher with higher infection intensity and were particularly higher at > 0.4 lice g^−1^ (Table 6).

**Table 5:** Specific growth rates (SGR, % day^−1^, Days 0 to 30), change in condition factor (ΔCF, Days 0 to 30) and condition factors (CF, day 30) in Atlantic salmon (*Salmo salar*) postsmolts categorized according to infection intensity (lice g^−1^, day 30)

**II (lice g** ^**−1**^ **)**	**Fish N** ^*^	**Mortality (%) Day 30**	**SGR (% day** ^**−1**^ **)**	**ΔCF**	**CF**
0 (control)	146	0	0.95	0.08	1.22
0.07–0.2	19	0	0.81	0.07	1.20
0.2–0.3	31 (1)	3	0.74 (−0.20)	0.05 (−0.18)	1.18 (1.01)
0.3–0.4	22 (2)	8	0.50(−0.01)	0.01 (−0.17)	1.15 (1.01)
0.4–0.5	24 (2)	8	0.34 (−0.40)	−0.03 (−0.10)	1.10 (1.00)
0.5–0.6	10 (3)	23	0.22 (0.07)	−0.08 (−0.05)	1.07 (1.07)
≥0.6	10 (4)	29	−0.04 (0.11)	−0.06 (−0.02)	1.06 (1.07)

Category ‘0’ are the uninfected control fish. The infected fish were infected with salmon lice (*Lepeophtheirus salmonis*) copepodids on Day 2, and number of lice counted on Day 30. Calculation of mortality is based on moribund and dead fish on Day 30.

Numbers in brackets are values from moribund fish.

**Table 6:** Plasma Cl^−^ (mmol L^−1^), Na^+^ (mmol L^−1^), osmolality (mOsm kg^−1^) and cortisol (ng ml^−1^) in Atlantic salmon (*Salmo salar*) postsmolts categorized according to infection intensity (lice g^−1^, Day 30)

**II (lice g** ^**−1**^ **)**	***N***	**Cl** ^**−**^	**Na** ^**+**^	**Osm**	**Cortisol**
0 (control)	45	140	169	347	86
0.07–0.2	4	145	169	343	80
0.2–0.3	9 (1)	149 (218)	175 (241)	352 (na)	72 (438)
0.3–0.4	8 (2)	162 (231)	182 (253)	377 (502)	159 (529)
0.4–0.5	8 (2)	191 (218)	213 (244)	411 (493)	321 (442)
0.5–0.6	5 (1)	187 (202)	211 (226)	400 (451)	293 (363)
≥0.6	1 (4)	191 (205)	207 (222)	447 (453)	510 (491)

Category ‘0’ are the uninfected control fish. The infected fish were infected with salmon lice (*Lepeophtheirus salmonis*) copepodids on Day 2, and number of lice counted on Day 30. *N* = number of fish. Numbers in brackets are values for moribund fish.

Infection intensity was significantly related with CF, SGR, ΔCF and plasma Na^+^, Cl^−^, osmolality and cortisol (Table 7). Using the regression equation from the simple regression between infection intensity (II, x-axis) and SGR (y-axis) (*R*^2^ = 0.36, *P* < 0.00001, *y* = 1.0381–1.4393^*^x), zero growth (SGR = 0) equalled an infection intensity of 0.72 lice g^−1^.

**Table 7:** Coefficient of determination (*R*^2^) and *P* values for the simple regressions between infection intensity (II, lice g^−1^, Day 30) and CF (day 30), SGR (% day^−1^, Days 0 to 30), ΔCF (Days 0 to 30) and plasma Na^+^, Cl^−^, osmolality and cortisol (day 30). ‘+’ indicate a positive relationship, while ‘−’ indicate a negative relationship

**Correlation**	***R*** ^**2**^	***P* value**	***N***
II vs CF	0.29 (−)	***<0.00001***	116
II vs SGR	0.36 (−)	***<0.00001***	116
II vs ΔCF	0.28 (−)	***<0.00001***	116
II vs Na^+^	0.42 (+)	***0.00003***	35
II vs Cl^−^	0.47 (+)	***0.00001***	35
II vs osmolality	0.37 (+)	***0.0001***	35
II vs cortisol	0.42 (+)	***0.00002***	35

Number in italic and bold indicates a significant correlation.

Do not include values from moribund fish.

Calculated infection intensity threshold values for response in plasma parameters were 0.18 lice g^−1^ for plasma Cl^−^ (Fig. 1A), and 0.22 lice g^−1^ for plasma Na^+^ (Table 8). When removing data from infection intensities < 0.55 lice g^−1^ (mean level on dead fish) (*n* = 3, 0.57, 0.58, 0.90 lice g^−1^), the determination coefficients for the infection intensity vs plasma simple regressions increased (Cl^−^, *R*^2^ = 0.64 (Fig. 1B); Na^+^, *R*^2^ = 0.59), but the calculated infection intensity threshold values for response in plasma ions showed minor changes (Cl^−^ increased from 0.18 to 0.22, and Na^+^ increased from 0.22 to 0.25).

**Table 8:** Calculated threshold infection intensity (TII, lice g^−1^) values for response in plasma Na^+^ and Cl^−^

**Simple regression**	**Regression equation**	**Control Mean + 2STDEV**	**TII (lice g** ^**−1**^ **)**
II vs Na^+^	*y* = 156.2286 + 92.7248^*^x	Plasma Na^+^: 176.3 (mmol L^−1^)	0.22
II vs Cl^−^	*y* = 129.3102 + 103.2867^*^x	Plasma Cl^−^: 147.5 (mmol L^−1^)	0.18

The simple regression equation (*y* = *a* + *bx*) between infection intensity (II, *x*-axis) and plasma Na^+^ and Cl^−^ (*y*-axis) from the infected group are based on *N* = 35 (not including moribund fish). Control group *N* = 45. The calculated threshold TII’s are based on mean plus two standard deviations plasma Na^+^ and Cl^−^ values from the control group

## Discussion

The present study defines the lice intensity laboratory threshold values for responses above the control level in plasma Na^+^, and Cl^−^ in 40 g Atlantic salmon postsmolts 28 days post infection at 9°C. This is vital knowledge for conservation practitioners wanting to understand the physiologically derived burden salmon lice can have on Atlantic salmon populations and can be used in the monitoring and conservation of Atlantic salmon populations affected by aquaculture-driven salmon lice infestations.

With the present 69% infection success, it is unlikely that our calculated threshold values are too conservative. Hence, the present threshold values should be appropriate for natural size Atlantic salmon postsmolts at 9°C under laboratory conditions. However, plasma ions and cortisol have not been studies in wild caught salmon lice infected Atlantic salmon postsmolts. Further, it is unknown if temperature affects the host response to salmon lice. The mean seawater temperature when salmon smolts migrate in May varies between 5 and 11°C depending on location along the Norwegian coast (http://www.imr.no/forskning/forskningsdata/stasjoner/view/initdownload). Hence, the present temperature at 9°C is relevant.

The lice induced osmotic stress appears as the lice matures on the fish, and plasma ions increases when the lice reach the pre-adult I stage in Atlantic salmon (Grimnes and Jakobsen, 1996; Finstad *et al*., 2000; Bowers *et al*., 2000) and sea trout (Wells *et al*., 2006). Lice induced mortalities started at 22 days post-infection, coinciding with the estimated development of pre-adult I stage male lice (Hamre *et al*., 2019). When the lice reach this stage of development, onset of deaths are known to occur in experimental infection challenges, and an extension of the experimental period would have increased mortalities in infected fish (Grimnes and Jakobsen, 1996; Bjørn and Finstad, 1998). At 28 days post infection, moribund fish occurred at infection intensities above 0.2—occurrence increasing with raising infection intensity—and all had extremely high plasma ion and cortisol levels. This is in line with earlier reports of extremely high plasma ion levels in moribund salmon lice infected Atlantic salmon (Grimnes and Jakobsen, 1996; Finstad *et al*., 2000), and more lice on dead fish (Finstad *et al*., 2007). The current correlations between plasma ions and cortisol in the lice infected fish may indicate that salmon lice induced a stress response in the host, which in turn caused elevated membrane permeability, osmotic imbalance and ultimately death. On the contrary, elevated plasma cortisol may be a protective mechanism to restore osmotic homeostasis in the host. Key component for osmoregulation is the branchial sodium–potassium pump (Na+/K+-ATPase; NKA) localized in the basolateral membrane of mitochondrial rich chloride cells in the gills (Marshall, 2002). In Atlantic salmon, cortisol up-regulates branchial NKA enzyme activity (McCormick *et al*., 2008), and salmon lice infection upregulates gill NKA enzyme activity (Nolan *et al*., 1999) and stimulates chloride cell proliferation (Farrell, 2011). Hence, the lice induced elevation of plasma cortisol may be a pure stress response and/or a protective mechanism to maintain ionic homeostasis by increasing branchial NKA enzyme activity. If it is a protective mechanism, lice-induced cutaneous lesions are the main cause for the hosts elevated plasma ions. It is unknown if there is a relationship between plasma cortisol level and gill NKA enzyme activity in salmon lice infected Atlantic salmon, and if other organs involved in osmoregulation, like the intestine and kidney, respond to salmon lice infection. Further, the lice infection intensity thresholds for responses in the osmoregulatory mechanisms need to be identified. If these thresholds are lower than those required for responses in plasma ions and cortisol, that would indicate that salmon lice cause ion leakage in the host through both cutaneous lesions and a stress response. The extremely high plasma ion levels in moribund fish would suggest a double negative action of salmon lice on the hosts plasma ion balance.

The present correlations between SGR and plasma Na^+^, Cl^−^, osmolality and cortisol in the infected fish are new knowledge and shows that lice-induced stress and/or osmoregulatory failure have a strong impact on growth. This can be related to reduced appetite and/or increased energy demand for osmoregulation. Growth of surviving lice-infected fish concerns the conservation of wild Atlantic salmon. Adult salmon lice are often observed on adult river running Atlantic salmon. If these salmon lice attached as copepodids during smolt migration is unclear, but laboratory studies have shown that salmon lice can survive on Atlantic salmon for over 1 year (Hamre *et al*., 2009). In accordance with the present laboratory study, a field study on river running wild Atlantic salmon in England and Scotland showed a correlation between number of infective salmon lice and body condition (Susdorf *et al*., 2018a). Indeed, models have shown that sea lice-mediated changes in body condition can cause population declines in wild Atlantic salmon due to changes in marine survival, fecundity and age at sexual maturation (Susdorf *et al*., 2018b). Whether the changes in body condition observed in lice-infected river running Atlantic salmon (Susdorf *et al*., 2018a) are related to chronic stress and/or osmoregulatory problems caused by lice infection during seaward migration is unknown and deserves further investigation.

Based on mean plus two standard deviation plasma values of the control fish, lice intensity threshold values were calculated to be 0.2 lice g^−1^ for plasma Cl^−^ and Na^+^. This is lower than reported in sea trout (Wells *et al*., 2006) and Arctic char (Fjelldal *et al*., 2019), where 0.3 lice g^−1^ is the reported threshold for physiological consequences. The national surveillance program of salmon lice on wild salmonids (NALO) catches wild salmonids using traps or nets along the Norwegian coast yearly (method described in Serra-Llinares *et al*., 2014). Of the 2501 wild Atlantic salmon postsmolts captured in 2019 in the NALO project (Nilsen *et al*., 2019), overall 31% were infected with salmon lice. The data are based on totally 40 samplings divided by week number and site. Totally, seven regions along the Norwegian coast were investigated, with number of fish captured per region ranging between 27 and 593. The average fish size and occurrence of lice infected fish ranged between 16 and 25 g, and 12 and 84%, respectively, between regions. Estimated lice infection intensities of infected fish, calculated using the reported lice infection level (lice per infected fish) and fish weight (including both infected and uninfected), ranged from 0.06 to 0.67 lice g^−1^ between regions. Two regions had estimated infection intensities greater than 0.5 lice g^−1^ (0.51 and 0.68 lice g^−1^), while five regions had estimated infection intensities below 0.2 lice g^−1^ (0.06–0.19 lice g^−1^). Hence, it is likely that salmon lice had an impact on plasma Na^+^ and Cl^−^, growth and mortality of wild Atlantic postsmolts in certain regions of Norway in 2019.
